# Purinergic receptor antagonism reduces interictal discharges and rescues cognitive function in a mouse model of temporal lobe epilepsy

**DOI:** 10.3389/fnins.2025.1513135

**Published:** 2025-04-04

**Authors:** Nelson Espinosa, Soraya Martín-Suárez, Ariel Lara-Vasquez, Trinidad Montero, Teresa Muro-García, German Fernandez, Juan Manuel Encinas-Pérez, Pablo Fuentealba

**Affiliations:** ^1^Departamento de Psiquiatria, Escuela de Medicina, Pontificia Universidad Católica de Chile, Santiago, Chile; ^2^Achucarro Basque Center for Neuroscience, Leioa, Bizkaia, Spain; ^3^IKERBASQUE, Basque Foundation for Science, Bilbao, Bizkaia, Spain; ^4^Department of Neurosciences, Faculty of Medicine and Nursing, University of the Basque Country, Leioa, Bizkaia, Spain

**Keywords:** purinergic receptor, temporal lobe epilepsy, interictal discharges, cognitive dysfunction, cortical oscillation

## Abstract

Epilepsy is one of the most prevalent neurological disorders globally. Current treatments mainly target neuronal activity, often overlooking the involvement of astrocytes and microglia in epilepsy’s pathophysiology. Here, we explored the impact of purinergic receptors, predominantly found in glial tissue, on epileptiform activity. We used TNP-ATP, a potent purinergic receptor antagonist, and conducted experiments using a mouse model of mesial temporal lobe epilepsy to examine behavioral performance and neural activity patterns. Our findings reveal that although TNP-ATP treatment did not significantly impact motor function or anxiety levels, it reduced both the amplitude and rate of hippocampal interictal discharges. Such reduction also affected the synchrony of associated neuronal spiking. Additionally, cognitive function, particularly hippocampus-dependent spatial memory and prefrontal cortex-dependent executive control, were partially restored. Moreover, neuronal recordings showed increased phase coherence between the hippocampus and prefrontal cortex for both slow (theta) and fast (gamma) oscillations in treated animals, indicating strengthened neural coordination between cortical regions upon purinergic receptor antagonism. These results underscore the potential role of purinergic receptor antagonists in improving behavioral and cognitive performance in epilepsy, providing novel insight into the use of these pharmacological agents as a therapeutic approach.

## 1 Introduction

Epilepsy is a major neurological disorder affecting millions of people worldwide, with a noticeably higher prevalence in low- and middle-income countries (Thijs et al., 2019; Beghi, 2020). Characterized by its diverse seizure types, ranging from minor absences to severe generalized tonic–clonic seizures, epilepsy arises from imbalances in excitatory and inhibitory neurotransmitters in the brain, mainly glutamate and gamma-aminobutyric acid (GABA), respectively (Lévesque and Avoli, 2013; Akyuz et al., 2021). This imbalance leads to neuronal hyperexcitability and hypersynchronized electrical brain activity, manifesting as electrographic seizures.

The primary treatment for epilepsy involves the use of antiseizure medications, yet their effectiveness is limited, highlighting the need for novel pharmacological strategies (Löscher and Schmidt, 2011). Recent advances in understanding epilepsy have drawn attention to the roles of astrocytes and microglia in its pathophysiology (Devinsky et al., 2013; Bedner and Steinhäuser, 2016). Indeed, during epileptic episodes, the release of glutamate and ATP activates purinergic receptors, which are prevalent on neurons, astrocytes, and especially microglia (Vezzani et al., 2019). Such activation triggers inflammatory responses via the release of pro-inflammatory cytokines (Avignone et al., 2008; Beamer et al., 2017).

P2X receptors (P2XRs), a subtype of purinergic receptors, are notably upregulated in microglia following seizures, suggesting a key role in epilepsy’s progression (Beamer et al., 2017). For example, P2X7R antagonists have shown potential in modulating epileptiform activity, probably enhancing the effects of traditional antiepileptic drugs (Amhaoul et al., 2016; Beamer et al., 2017). The activation of ATP receptors, particularly the P2X7R, is increasingly recognized as a crucial factor in controlling brain hyperexcitability, inflammation, and potentially offering neuroprotection (Beamer et al., 2017; He et al., 2017).

Despite the expanding body of research, further study is necessary to establish the precise role of ATP signaling in epilepsy. ATP, often co-stored with classic neurotransmitters like GABA and glutamate, shows a significant increase in extracellular levels during pathological conditions (Illes et al., 2019). The rise in extracellular ATP levels is known to activate P2XRs on brain immune cells, exacerbating neuroinflammation, a key factor in the progression of epilepsy (Skaper et al., 2010). Given this, targeting ATP receptors, especially P2XRs, has been hypothesized to be instrumental in modulating neuronal excitability, neuroinflammation, and neuroprotection. Hence, we explored the therapeutic potential of targeting ATP receptors in epilepsy treatment, with a specific interest on TNP-ATP, a purinergic receptor antagonist, in a mouse model of mesial temporal lobe epilepsy. We concentrated on behavioral performance, cognitive function, and neural activity patterns, as these features remain little explored in preclinical models. We tested the hypothesis that purinergic receptor antagonism reduces interictal discharges and rescues cognitive function in a mouse model of low-level temporal lobe epilepsy. By examining both the behavioral and neural impacts of this treatment, our research contributes to substantiate the use of purinergic antagonists in epilepsy’s pathophysiology and potential new treatment avenues.

## 2 Methods and methods

### 2.1 Animals

A total of 18 male mice were used in this study. Mice were housed in a temperature- and humidity-controlled room (22 ± 2°C) with food and water available ad libitum. A group of C57BL/6J mice (*n* = 10) were used for electrophysiological and behavioral experiments, whereas another group of Nestin-GFP mice (*n* = 8) were used for immunofluorescent experiments. Nestin-GFP mice, used for immunostaining experiments for astrogliosis, were kindly provided by Dr. Grigori Enikolopov at Cold Spring Harbor Laboratory (Cold Spring Harbor, NY, USA). These mice were crossbred with C57BL/6 mice for at least 10 generations (Mignone et al., 2004) and were kept at the animal house facilities at the UPV/EHU campus in Leioa, Spain in standard conditions of housing. All procedures were approved by the University of the Basque Country (UPV/EHU) Ethics Committees (Leioa, Spain) and Diputacio Foral de Bizkaia under protocol M20-2022-130 and M30-2022-129. All procedures followed the European directive 2010/63/UE and National Institutes of Health guidelines. Efforts were made to minimize the number of animals used and their suffering.

### 2.2 5-Bromo-2′-deoxyuridine (BrdU) administration

BrdU [5-Bromo-1-(2-deoxy-*β*-D-ribofuranosyluracil, 5-Bromouracil deoxyriboside); Sigma, St Louis, MO, USA] was diluted in sterile saline and administered through intraperitoneal injections at 150 mg/kg concentration (15 mg/mL of BrdU was diluted in sterile phosphate-buffered saline (PBS) with 0.01 N sodium hidroxyde (1% of the total solution). BrdU was administered intraperitoneally (3 injections, 24 h-apart) on the 3 last days of TNP-ATP (or vehicle) administration.

### 2.3 Immunohistochemistry

Mice were sacrificed 21 days after the KA injection. Experiments were performed essentially as described before following methods optimized for the use in transgenic mice (Encinas et al., 2006, 2011; Encinas and Enikolopov, 2008). Animals were deeply anesthetized and were subjected to transcardial perfusion with 25 mL of PBS followed by 30 mL of 4% (w/v) paraformaldehyde in PBS, pH 7.4. The brains were removed and post-fixed, with the same fixative solution, for 3 h at room temperature, then transferred to PBS and kept at 4°C. Quantitative analysis of cell populations in transgenic mice was performed by means of design-based (assumption free, unbiased) stereology using a modified optical fractionator sampling scheme as previously described (Encinas and Enikolopov, 2008). Slices were collected using systematic-random sampling. The right hemisphere was selected per animal. The hemisphere was sliced sagittally in a lateral-to-medial direction, from the beginning of the lateral ventricle to the middle line, thus including the entire dentate gyrus. We focused on the dentate gyrus because neurogenesis is well-documented in the subgranular zone, and this region is particularly vulnerable to epileptic insults. The 50 μm slices (cut using a Leica VT 1200S vibrating blade microtome, Leica Microsystems GmbH, Wetzlar, Germany) were collected in 6 parallel sets, each set consisting of 12 slices, each slice 300 μm apart from the next. The sections were incubated with blocking and permeabilization solution (PBS containing 0.25% Triton-100X and 3% BSA) for 3 h at room temperature, and then incubated overnight with the primary antibodies (diluted in the same solution) at 4°C. After thorough washing with PBS, the sections were incubated with fluorochrome-conjugated secondary antibodies diluted in the blocking and permeabilization solution for 3 h at room temperature. After washing with PBS, the sections were mounted on gelatin coated slides with DakoCytomation Fluorescent Mounting Medium (DakoCytomation, Carpinteria, CA). Those sections destined to the analysis of BrdU incorporation were treated, before the immunostaining procedure, with 2 N HCl for 20 min at 37°C, rinsed with PBS, incubated with 0.1 M sodium tetraborate for 10 min at room temperature, and then rinsed with PBS. The GFP signal from the transgenic mice was detected with an antibody against GFP for enhancement and better visualization. The following antibodies were used: chicken anti-GFP (Aves Laboratories, Tigard, OR) at 1:1000 dilution; rabbit anti-S100β (Dako Cytomation) at 1:1000 and rat anti-BrdU (AbD Serotech, Kidlington, UK) at 1:1000. As secondary antibodies, AlexaFluor 488 goat anti-chicken (Molecular Probes, Willow Creek Road, Eugene, OR) at 1:500; AlexaFluor 647 goat anti-rabbit (Molecular Probes) at 1:500; AlexaFluor 568 goat anti-rat (Molecular Probes) at 1:500 were used. In randomly selected fields, at 63x, (by starting at the lateral tip of the upper blade of the GCL and then skipping 2 field and imaging the 3rd, the 6th etc.), following the GCL (upper and lower blade) along the dentate gyrus, 50–100 BrdU cells per animal were categorized as astrocytes, or reactive astrocytes following the criteria described previously (Encinas et al., 2006; Encinas and Enikolopov, 2008; Sierra et al., 2015). Astrocytes were identified as S100β-positive cells, negative for Nestin-GFP staining, with stellar morphology. Reactive astrocytes were defined as Nestin- GFP-positive cells immunopositive also S100β and with hypertrophic soma, thickened processes and star-like morphology. Those cells in the uppermost focal plane were not counted to avoid overestimation. The total number of astrocytes and of reactive astrocytes was estimated quantifying their number in the granule cell layer and hilus in one set of slices and then multiplying by 6.

### 2.4 Microdrive construction

Custom-made microdrives carrying 6 electrodes (Microprobes) were assembled for simultaneous bilateral electrophysiological recording of local field potentials in the prefrontal cortex and the dorsal CA1 area of the hippocampus. One electrode was inserted in the bundle targeting the prefrontal cortex, and two electrodes in the bundle targeting the hippocampus. Each electrode was cemented to a single fixed bundle and was connected to an 8-channel printed circuit board assembled to an Omnetics connector.

### 2.5 Stereotaxic surgery

Animals aged 60 days were used for implantation of microdrives. Mice were initially anesthetized with isoflurane (4% isoflurane with 100% O_2_) before being placed in a stereotaxic frame, and anesthesia was maintained using isoflurane (1–2% isoflurane with 100% O_2_) until the surgery was finished. Rectal temperature was monitored, and core temperature (37°C) was maintained with a homeothermic blanket. After incision in the scalp, two burr holes were drilled at stereotaxic coordinates targeting the prefrontal cortex (1.94 mm AP, 0.5 mm ML, from Bregma) and the CA1 region of the hippocampus (−1.54 mm AP, 1.5 mm ML from Bregma). We targeted the CA1 area because, as the main output region of the hippocampus, it establishes strong functional connectivity with the frontal cortex that is crucial for spatial navigation, memory formation and sleep coordination (Siapas and Wilson, 1998; Negrón-Oyarzo et al., 2018). The dura was removed, and a glass pipette was descended through the brain (2 mm DV) to inject KA directly on the hippocampus. After injection, the pipette was removed and the recording electrodes were lowered to the cortical surface. Two ground wires were attached to skull screws, and the microdrive was affixed to the skull with dental acrylic. After surgery, animals were maintained in individual cages in a room with controlled temperature (22 ± 2°C) and humidity, with food and water available ad libitum. Mice were allowed to recover for 1 week after surgery before beginning behavioral experiments and electrophysiological recordings. During recovery, weight and general health were monitored daily, and animals received an intraperitoneal dose of analgesic (Ketoprofen, 5 mg/kg/day) and antibiotic (Enrofloxacin, 5 mg/kg/day). One day before surgery, mice were I.P. injected with either saline (i.e., control) or TNP-ATP (i.e., treated, 10 mg/kg). The procedure was then repeated for 7 days after surgery.

### 2.6 Electrophysiological recordings

After a week of recovery from surgery, mice were placed in a custom-built booth, and microdrives were connected to a headstage (model RHD2116 Intan Tech, CA, USA). Neural signals were amplified (200 times), digitized (sampled at 20 kHz), filtered (0.5–5,000 Hz), and monitored through an amplifier board (RHD2000 evaluation system; Intan Tech, CA, USA).

### 2.7 Behavioral experiments

For the probabilistic feeding task, animals were exposed to a FED3 [feeding experiment device (Nguyen et al., 2016)] that dispensed a pellet (sweet, popped quinoa) from the central port every time the animal nose-poke on one of the lateral ports. The probability of receiving a pellet was larger in one lateral port (80 vs. 20%), and after a variable number of trails (15 ± 2), the rule was reversed. Both the operant behavior (video tracking) and the electrophysiological activity were simultaneously recorded for 10 min. For the metric spatial change task, animals underwent 4 sessions in a rectangular arena (50 cm × 30 cm × 30 cm high), each lasting 5 min. During the first 3 sessions, animals were allowed to explore two objects placed 20 cm apart and positioned 5 cm from the arena walls, with a 3-min intersession interval. In the last session (test), following a 10-min intersession interval, the objects were repositioned so that the separation between them was reduced to 10 cm (Goodrich-Hunsaker et al., 2008). Importantly, the exploration time of individual objects was not measured. Instead, the total exploration time for both objects combined was calculated and compared between the sample and test conditions.

### 2.8 Cross-frequency modulation

Phase-amplitude cross-frequency coupling modulation index for several frequency pairs of theta-band “phase-modulating” and 2–200 Hz “amplitude-modulated” components was evaluated. We first filtered spectral components of the LFPs in the theta frequency (4–8 Hz) and amplitude bandwidth 4 Hz at 2-Hz-steps were used to obtain the amplitude between 2 and 200 Hz. We next calculated the Hilbert transform to obtain the instantaneous phase of theta oscillation and the instantaneous wideband amplitude oscillations. The modulation index was obtained by means a normalized entropy index described in Tort et al. (2008).

### 2.9 Cross-correlations

Interictal discharge onset was defined with point 0 to correlate both hippocampal neural activity and contralateral ID onset by applying the “sliding-sweeps” algorithm. The timestamps of contralateral interictal discharges and hippocampal spikes within a time window (0.5 and 1.5 s respectively) were considered as a template and were represented by a vector of spikes relative to *t* = 0 s, with a time bin (100 and 200 ms respectively). Next, the window was shifted to successive interictal discharges onset throughout the recording session, and an array of recurrences of templates was obtained. Cross-correlations were obtained by averaging the array and normalizing to the basal activity.

### 2.10 Granger causality

The multivariate Granger causality (MVGC) Matlab toolbox was used to assess pairwise causalities between LFP–LFP activity. This toolbox, available online,^1^ allows a fast and accurate estimation of the Wiener–Granger causal inference in the frequency domain. Estimators were calculated with the standard ordinary least squares and with a model order of 50. Frequency resolution was set at 1000.

### 2.11 Spectrograms

Time-frequency spectra for LFP recordings were computed by a wavelet time frequency transformation (using Morlet wavelets) based on convolution in the time domain and using the FieldTrip function ft_freqanalysis.m.^2^

### 2.12 Histology

After completion of experiments, mice were anesthetized with isoflurane (3%), and then transcardially perfused with 20 mL of saline solution followed by 50 mL of 4% paraformaldehyde in phosphate-buffered saline (PBS, pH 7.4). The brain was removed, incubated overnight in 4% paraformaldehyde in PBS, and then stored in PBS containing 0.2% sodium azide. Coronal brain slices (60 μm) were prepared from paraformaldehyde-fixed brains with a vibratome (World Precision Instruments, Sarasota, USA) in ice-cold PBS. For visualization of the electrolytic lesions, slices were stained with Nissl staining, and images were acquired with a microscope (Nikon).

### 2.13 Locomotor patterns

DeepLabCut, an open-source video tracking system, was used to automatically collect the mouse’s instantaneous position along the track. The animal trajectory was smoothed (mobile mean of 10 successive points) to avoid artifacts due to space discretization, and the animal’s instantaneous speed was quantified as the mean displacement between adjacent frames divided by the frame time (camera acquisition at 30 fps).

### 2.14 LFP spectral power and coherence

For analysis of power of oscillatory activity, electrophysiological recordings were downsampled to 1,000 Hz, bandpass-filtered at 0.1–100 Hz, and power spectral density (PSD) and coherence were computed using multitaper Fourier analysis from the Chronux toolbox.^3^ Interictal discharges were detected by high pass filtering (>40 Hz; Butterworth, order = 4) the CA1 LFP and the z-scored squared signal was calculated. Peaks above 5 standard deviations were identified as putative interictal discharges. For PSD analysis, field potentials were divided into 5,000 ms segments with 500 ms overlap, and a time-bandwidth product (TW) of 3 and 5 tapers. For spectral coherence, field potentials were divided into 2000 ms segments with 100 ms overlap, and a time-bandwidth product (TW) of 5 and 9 tapers. Mean spectral power and coherence measures were calculated for theta (6–10 Hz), slow-gamma (20–40 Hz), and fast-gamma (60–80 Hz) bands for the entire trial session, and separately for start, navigation, and escape phases of the task.

### 2.15 Spike sorting

Spike sorting was performed offline using MATLAB-based graphical cluster-cutting software, Mclust/Klustakwik toolbox (version 3.5). To detect spikes, broadband recordings sampled at 20 kHz were filtered at 600–5000 Hz, and events with amplitudes reaching 5–50 standard deviations over the mean were considered as candidate spikes. For each recording file, single channels from individual tetrodes were identified, and a single file was generated per channel. Then, generated files from the same electrode were clustered by Principal Components Analysis (PCA) using MClust 3.5 toolbox running on MATLAB. The spike features considered for clustering included peak amplitude (the maximum height of the waveform of each channel of each spike), timestamp (the time of occurrence of each spike), spike width (the duration in each channel of each spike), energy (the energy contained within the waveform of each channel of the spike), valley (the maximum depth of the waveform of each channel of the spike), and wave-PC (the contribution to the waveform due to the principal component). MClust automatically identified similar clusters by assigning values between 0.0–1.0 for each cluster, in which similar clusters displayed values close to 1.0. Similarity between clusters was manually confirmed through visual inspection of spike features. Final confirmation was done by examining the ISI for every single unit and establishing that no spikes were present at ISIs shorter than 2 ms. Finally, a file for every single unit including timestamps of spikes for every tetrode was generated and exported, and this file was used in the subsequent analysis on MATLAB.

### 2.16 Statistical analysis

Comparison between behavioral parameters (latency time, errors) and other normally distributed parameters were analyzed with parametric analysis (t-student test; one-way ANOVA followed by Bonferroni post-hoc test). Comparison between firing rates and other non-normally distributed parameters were analyzed with non-parametric tests (Mann–Whitney U-test; Kruskal-Wallis test followed by Dunn’s multiple comparison post-hoc test). Linear correlations between parameters were analyzed by Spearman correlation test. Comparisons between the slopes of significant linear regressions were performed using a bootstrap resampling method with replacement and testing the differences between the distributions of the slopes over 1,000 iterations. Comparisons between PETHs were analyzed with the Wilcoxon test. Statistical analysis was performed with GraphPad Prism software or with MATLAB (The MathWorks Inc.). Significant differences were accepted at *p* < 0.05. Summary statistical results are presented in Supplementary Table 1.

## 3 Results

We injected a single dose of kainic acid (KA, 50 nL, 2.22 mM) in the right dorsal CA1 area of the hippocampus in anesthetized mice to model early stages of mesial temporal lobe epilepsy development (Bouilleret et al., 1999; Sheybani et al., 2018). This is considered an intermediate dose, with a single injection effectively inducing mild non-convulsive seizures (Bielefeld et al., 2017), making it suitable for studying interictal activity and behavioral performance (Figure S1). Concurrently, during stereotaxic surgery, we implanted an electrode array targeting both the medial prefrontal cortex and dorsal CA1 area (Figure S2). Furthermore, mice were randomly injected the day before surgery with either saline (i.e.; control) or TNP-ATP (i.e.; treated), a potent P2XR blocker (North and Surprenant, 2000), to evaluate its effect on epileptiform activity and cognitive performance. To assess the effect of TNP-ATP, we quantified astrogliogenesis and astrogliosis in the hippocampus (Figure S3). Treated mice exhibited a reduced number of astrocytes and reactive astrocytes compared to control mice. Since the expression of nestin is a hallmark of reactive astrocytes, we used nestin-GFP transgenic mice for this experiment (Mignone et al., 2004). Astrocytes expressed GFAP but not nestin-GFP, while reactive astrocytes expressed both biomarkers (Figure S3). Hence, TNP-ATP reduced both astrogliogenesis and astrogliosis.

**Figure 1:**
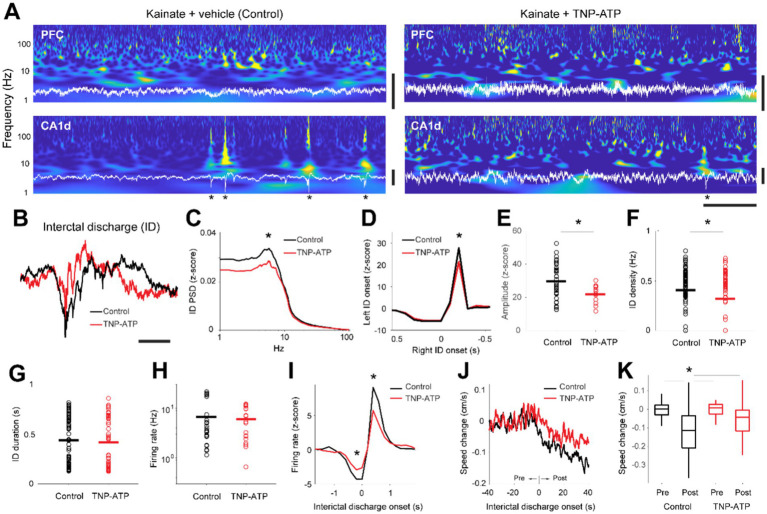
Interictal activity in the hippocampus of an epilepsy rodent model. **(A)** Spectrograms (blue) calculated from the local field potentials (white) recorded in the medial prefrontal cortex (PFC) and dorsal hippocampus (CA1), for animals injected with saline (left, kainite + vehicle) or a purinergic receptor antagonist (right, kainate + TNP-ATP). Asterisks depict interictal discharges in the dorsal hippocampus. Horizontal bar, 1 s; Vertical bars, 1 mV. **(B)** Overlay average interictal discharges (ID) recorded in the dorsal hippocampus from control (black) and TNP-ATP-treated animals (red). Scalebar, 200 ms. **(C)** Power spectral density of detected interictal discharges for control (black) and treated (red) animals. Power differed at peak frequencies (6 Hz) of interictal discharges (two-sided Wilcoxon rank-sum test, z = 2.4, *p* = 0.015). **(D)** Crosscorrelograms between interictal discharges detected on the right hemisphere (reference time) and left hemisphere. Note that interictal activity in the right hemisphere precedes left hemisphere discharges [unpaired t-test, *t*(47) = 2.2, *p* = 0.031, 27.7 ± 1.6 z-score, control; 21.4 ± 0.8 z-score, TNP-ATP]. **(E)** Amplitude of interictal discharges for control (black) and treated (red) animals (unpaired t-test, *p* = 0.012, 29.6 ± 1.6 z-score, control; 21.9 ± 0.9 z-score, TNP-ATP). **(F)** Density of interictal discharges for control (black) and treated (red) animals [unpaired t-test, *t*(158) = 2.3, *p* = 0.019, 0.41 ± 0.02 Hz, control; 0.32 ± 0.03 Hz, TNP-ATP]. **(G)** Duration of individual interictal discharges for control (black) and treated (red) animals [unpaired t-test, *p* = 0.64, *t*(120) = 0.46, 0.44 ± 0.03 vs. 0.42 ± 0.04 Hz]. **(H)** Average firing rates recorded from single units per session [unpaired t-test, *p* = 0.69, *t*(40) = 0.39, 6.7 ± 1.36 vs. 5.98 ± 0.96 Hz]. **(I)** Crosscorrelograms between interictal discharges and firing rates recorded from single units (before ID onset, unpaired t-test, *p* = 0.012, −4.3 ± 0.39 z-score, control; −2.9 ± 0.32 z-score, TNP-ATP; after ID onset, unpaired t-test, *p* = 0.012, 9.10 ± 0.95 z-score, control; 5.74 ± 0.80 z-score, TNP-ATP). **(J)** Average changes in locomotion speed during recordings of spontaneous behavior in reference to the onset of interictal discharges. **(K)** Box plots showing changes in locomotion speed preceding (pre) and following (post) the onset of interictal discharges. Note the ‘control post’ was larger than the other conditions. Asterisk depicts significant differences produced by TNP-ATP (one-way ANOVA, *p* = 1.34 × 10^−7^. *Post hoc* Tukey–Kramer’s multiple comparison test; control post vs. control pre, *p = 1.1 × 10^−7^; control post vs. TNP-ATP pre, *p* = 3.3 × 10^−6^; control post vs. TNP-ATP post, *p* = 0.03). Asterisks depict statistically significant differences.

Next, we evaluated epileptiform activity in control and treated animals. We identified interictal discharges by prominently large, rapid deflections in the local field potentials of the dorsal hippocampus (Figures 1A,B). The amplitude of interictal discharges was affected by TNP-ATP, as it was significantly decreased in treated animals when compared to control mice (two-sided Wilcoxon rank-sum test, *p* = 0.015, Figure 1C), consistently propagating from the injection site to the contralateral hemisphere (Figure 1D). Such propagation was slightly, though significantly, diminished by TNP-ATP (unpaired t-test, *p* = 0.012, Figure 1E). Importantly, high frequency activity (>40 Hz) was not different between treated and control animals (Figure S4), thus differences in baseline activity are unlikely to affect the proper determination and identification of interictal events. Furthermore, the incidence of interictal discharges was significantly diminished by TNP-ATP (unpaired t-test, *p* = 0.019, Figure 1F), particularly on the KA-injected hemisphere (unpaired t-test, *p* = 0.007). Conversely, TNP-ATP did not influence the duration of interictal discharges (Figure 1G), nor did it affect unit discharge (Figure 1H). However, we observed a significant decrease in the coupling of neuronal spiking to interictal discharges, as demonstrated by the crosscorrelogram between both activity patterns (unpaired t-test, *p* = 0.012, Figure 1I). During spontaneous behavior, animals in both control and treated groups explored and moved around the box at similar speeds (unpaired t-test, *p* = 0.58). However, the onset of interictal discharges triggered a consistent decrease in movement speed, thus altering movement patterns (Figure 1J). Interestingly, the speed decrease was slightly, though significantly, attenuated by TNP-ATP (one-way ANOVA, *p* = 1.34×10^−7^, Figure 1K). Collectively, these findings indicate that blocking P2XRs diminishes several key aspects of epileptiform activity in the temporal lobe.

**Figure 2:**
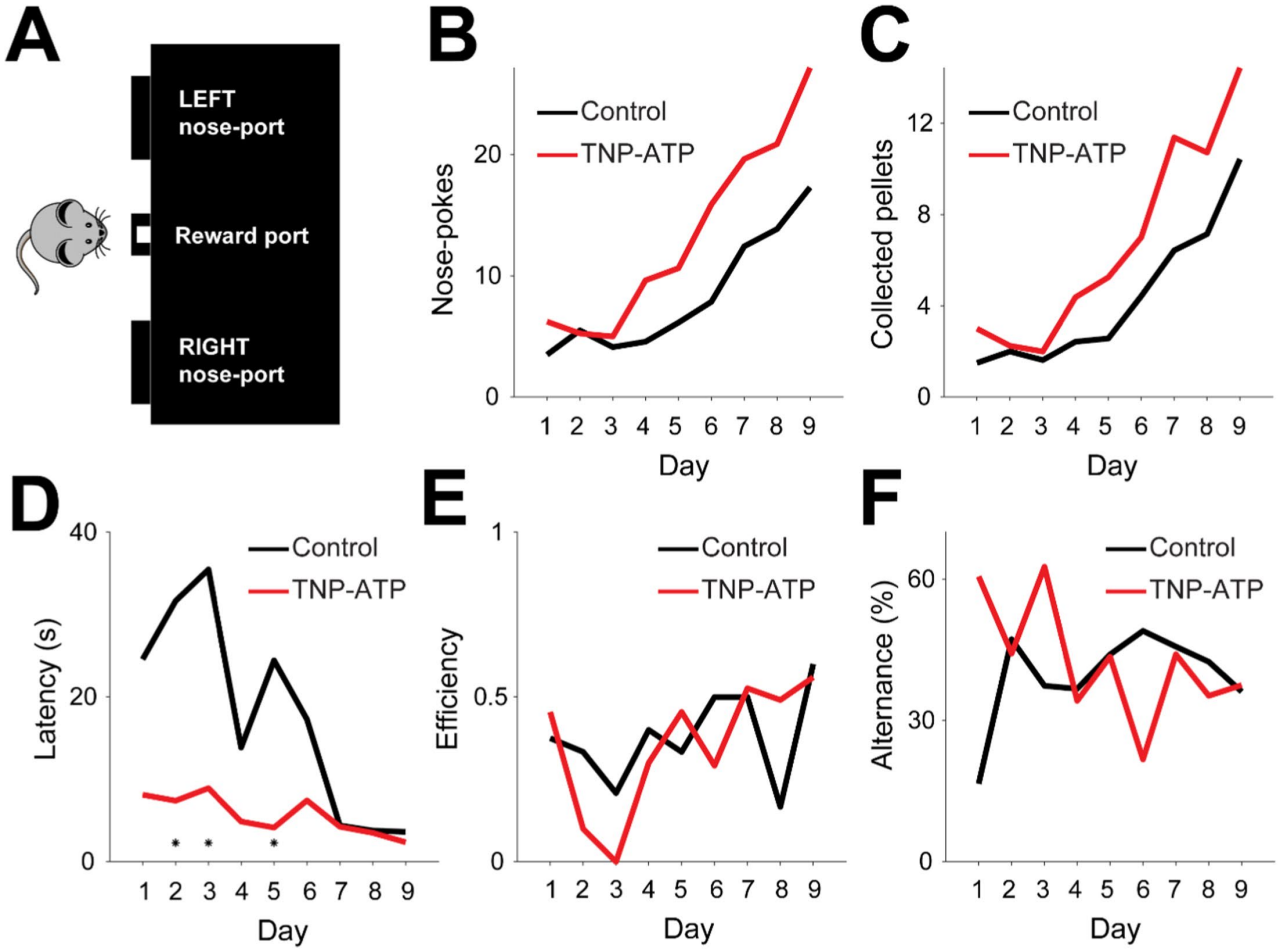
Behavioral performance during the probabilistic feeding task. **(A)** Diagram of the Feeding Experimentation Device (FED) used to implement the behavioral task. Mice were trained to nose poke on the lateral spouts to obtain reward (i.e., a small piece of food pellet) in the middle spout. Lateral spouts had fixed reward-delivering probabilities (80 and 20%), which were then reversed at the end of every training block (15 ± 2 trials). **(B)** Average number of nose pokes performed by mice in relation to training day. Both control and treated animals increased their number of nose pokes (Pearson correlations; control, *p* = 3.5×10^−4^; TNP-ATP, *p* = 7.5×10^−4^), yet the increase rate was significantly larger for TNP-ATP-injected animals (Bootstrap test for regression slopes, 100 iterations, *p* = 5×10^−4^; 1.67 ± 0.01 nose pokes/day, control; 2.77 ± 0.01 nose pokes/day, TNP-ATP). **(C)** Average number of collected reward by mice in relation to training day. Both control and treated animals increased the number of collected pellets, yet the increase rate was significantly larger for TNP-ATP-injected animals (Pearson correlation, control, *p* = 4.9×10^−5^; TNP-ATP, *p* = 1.3×10^−3^. Bootstrap test for comparing linear regression slopes, 100 iterations, *p* = 0.02, 1.05 ± 0.01 pellets/day, control; 1.55 ± 0.01 pellets/day, TNP-ATP). **(D)** Average latency to collect pellets from the middle nozzle by mice in relation to training day. Both control and treated animals progressively decreased their latency, yet it was significantly smaller for TNP-ATP-injected animals (Pearson correlation, control, *p* = 3.1×10^−3^; TNP-ATP, *p* = 0.013. Bootstrap test for comparing linear regression slopes, 100 iterations, *p* = 10^−4^, −0.68 ± 0.01 s/day, control; −3.87 ± 0.03 s/day, TNP-ATP). **(E)** Task efficiency in relation to training day. Efficiency was calculated as the ratio between the total number of collected pellets and total nose pokes (Pearson correlation, control, *p* = 0.35; TNP-ATP, *p* = 0.08). **(F)** Alternance proportion in relation to training day (Pearson correlation, control, *p* = 0.24; TNP-ATP, *p* = 0.09).

We then assessed cognitive function in the epilepsy model. For this, we took advantage of the feeding experimentation device [FED (Nguyen et al., 2016)], a programmable pellet dispensing machine that releases food with varying probability (Figure 2A). A pellet was delivered upon nose-poking with larger probability in one spout of the FED (80%), and after a variable number of trails (15 ± 2), the rule was reversed. In order to maximize the amount of collected reward in this probabilistic feeding task, mice had to acquire the reversal rule. During early stages of training, both groups exhibited similar nose poking behavior, which significantly increased after several days of training (Pearson correlation, *p* < 0.001, Figure 2B). However, the rate of nose-poking was larger in treated animals when compared to control mice (Bootstrap test, *p* < 3.1×10^−15^, Figure 2B), thus suggesting increased motivation in the task. Similarly, both groups significantly increased the collected reward during training (Pearson correlation, *p* < 0.01, Figure 2C). Nevertheless, treated animals collected pellets at a higher rate compared to control mice (Bootstrap test, *p* < 2×10^−18^, Figure 2C). Further, latency for pellet collection quickly decreased over sessions for both treated and control animals (Pearson correlation, *p* < 0.05, Figure 2D), thus suggesting that treated animals were faster to acquire and reinforce nose poking behavior. Moreover, treated animals decreased their latency at a faster rate compared to control mice (Bootstrap test, *p* < 10^−20^, Figure 2D). These findings suggest that treated mice displayed greater motivation and task engagement compared to control animals. However, the efficiency of pellet collection did not improve over time for either experimental group (Pearson correlation, *p* > 0.05, Figure 2E), suggesting that increased behavioral motivation did not reflect the acquisition of task-relevant rules. Indeed, alternance, the simplest behavioral strategy for pellet collection, did not change over time for either experimental group (Pearson correlation, *p* > 0.05, Figure 2F). Therefore, our results indicate that although cognitive flexibility remained unaffected, general task motivation and engagement improved in treated animals.

**Figure 3:**
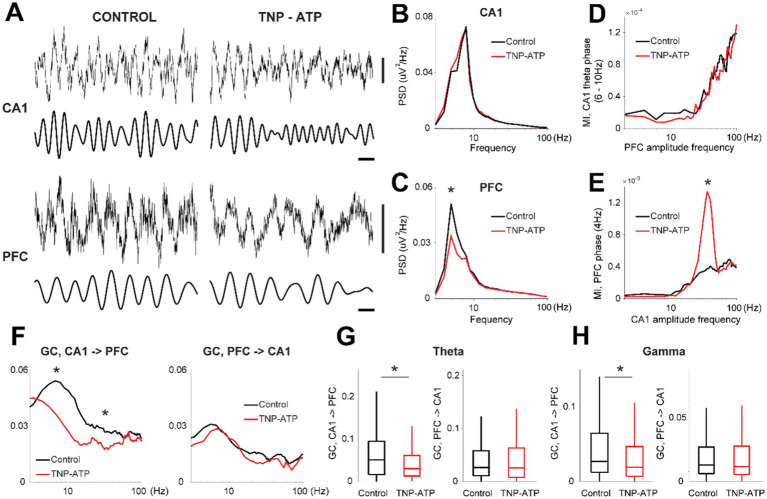
Oscillatory network activity during performance of a probabilistic feeding task. **(A)** Examples of cortical oscillations recorded during task performance. LFP (0.1–500 Hz), delta (3–4 Hz), theta (5–10 Hz). Horizontal bar, 200 ms; Vertical bars, 200 uV. Average power spectral distribution for LFP recording from hippocampus **(B)** and prefrontal cortex **(C)** for control and treated animals (two-sided Wilcoxon rank-sum test, *z* = 3.5, *p* = 4.0×10^−4^). Cross frequency modulation (MI, modulation index) between the phase of slow oscillations and the amplitude of fast oscillations for the hippocampus (**D**, theta) and prefrontal cortex (**E**, 4-Hz) for control and treated animals (two-sided Wilcoxon rank-sum test, *z* = −2.57, *p* = 0.01). **(F)** Granger causality for ascending (left, CA1 - > PFC) and descending (right, PFC - > CA1) drives for control and treated animals (two-sided Wilcoxon signed rank test with false discovery rate correction for multiple comparisons, *p* < 0.001). Box plot representation for the magnitude of Granger causality for theta (**G**, two-sided Wilcoxon rank-sum test, *p* = 3.3×10^−5^) and gamma (**H**, two-sided Wilcoxon rank-sum test, *p* = 8.2×10^−4^) frequency bands. Asterisks depicts significant differences.

We then assessed neural activity in the hippocampus and prefrontal cortex during the feeding task. Theta oscillations (4–10 Hz) dominated hippocampal activity during task performance in both control and treated animals (Figure 3A), which were not affected by TNP-ATP (two-sided Wilcoxon rank-sum test, *p* = 0.25, Figure 3B). In addition, the 4-Hz oscillation (Fujisawa and Buzsáki, 2011) was prominent in the prefrontal cortex, which was significantly decreased in treated animals (two-sided Wilcoxon rank-sum test, *p* = 6.4×10^−9^, Figure 3C). The power of faster activity patterns, like gamma oscillations (30–80 Hz), remained unaffected by purinergic blockade in both the hippocampus (two-sided Wilcoxon rank-sum test, *p* = 0.48) and prefrontal cortex (two-sided Wilcoxon rank-sum test, *p* = 0.99). No changes in cross frequency modulation were detected between the hippocampal theta rhythm and prefrontal cortex oscillations upon treatment (false discovery rate, *p* > 0.05, Figure 3D), yet the comodulation between slow 4-Hz cortical oscillations and gamma hippocampal oscillations was significantly enhanced by TNP-ATP (two-sided Wilcoxon rank-sum test, *p* = 0.01, Figure 3E). Moreover, we further investigated hippocampo-cortical interactions with Granger causality, which provides information about the directionality of connectivity between neural nodes (Friston et al., 2013). Hence, we found in control animals, and as previously reported (Broggini et al., 2016), a significant increase in ascending causality (i.e.; CA1 - > PFC) in both the theta and gamma bands during interictal intervals, whereas the descending drive (i.e.; PFC - > CA1) was not affected by TNP-ATP (false discovery rate, *p* > 0.05, Figure 3F). Moreover, the ascending drive was significantly reduced in treated animals, in both the theta range (two-sided Wilcoxon rank-sum test, *p* = 3.3×10^−5^, Figure 3G) and gamma band (two-sided Wilcoxon rank-sum test, *p* = 8.2×10^−4^, Figure 3H).

**Figure 4:**
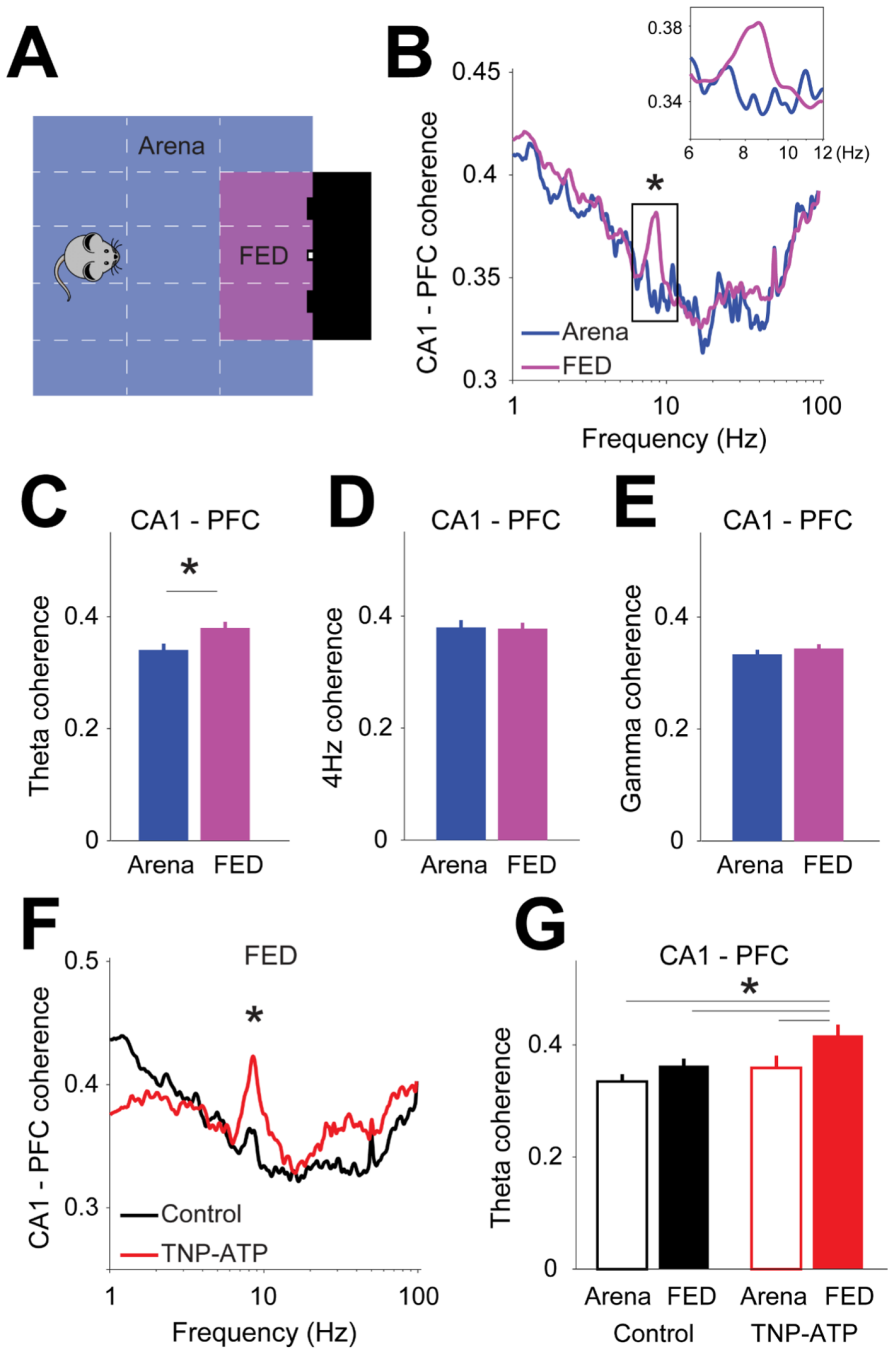
Cortical connectivity during performance of a probabilistic feeding task. **(A)** Definition of task-engaged (i.e., FED) and non-engaged (i.e., arena) zones during behavioral test. **(B)** Phase coherence between hippocampus and prefrontal cortex for task-engaged and non-engaged areas (one-sided Wilcoxon signed rank test with false discovery rate correction for multiple comparisons, *p* < 0.004). Inset depicts theta band phase coherence. Bar plots of phase coherence for theta (**C**, one-sided Wilcoxon signed rank test, *z* = −3.5, *p* = 4.6×10^−4^), 4-Hz (**D**, one-sided Wilcoxon signed rank test, *z* = −0.87, *p* = 0.38), and gamma (**E**, one-sided Wilcoxon signed rank test, *z* = −0.63, *p* = 0.53) frequency bands. **(F)** Phase coherence between hippocampus and prefrontal cortex for control and treated animals (two-sided Wilcoxon signed rank test with false discovery rate correction for multiple comparisons, *p* < 4.6×10^−3^). **(G)** Bar plots depicting theta oscillation phase coherence during task performance. Note blocking purinergic transmission increases coherence selectively during task-engaged states (two-way ANOVA: arena vs. FED, *p* = 0.02; control vs. TNP-ATP. Post-hoc Tukey–Kramer’s test to correct for multiple comparisons, *p* = 0.03; control arena vs. TNP-ATP FED, *p* = 8.9×10^−4^; control FED vs. TNP-ATP FED, *p* = 0.016; TNP-ATP arena vs. TNP-ATP FED, *p* = 0.002). Asterisks depict significant differences.

Next, we assessed hippocampo-cortical coordination during performance of the probabilistic feeding task. For this, we behaviorally separated states of task engagement (i.e., nose poking or pellet collection) from non-engaged states (i.e., arena exploration or grooming) (Figure 4A). That is, animals were considered as task-engaged only during direct interaction with the FED. Typically, during task-engaged states mice exhibited little displacement, whereas during non-engaged states animals moved more around in the arena (Figure S5). Therefore, to control for variations in movement patterns, we analyzed only those task episodes where the movement speed fell within two standard deviations from the average speed at the FED (Figure S5). We then calculated and compared the phase coherence between field potentials recorded in the hippocampus and prefrontal cortex during task performance (one-sided Wilcoxon signed rank test, *p* < 0.004, Figure 4B) and found coupling in the theta band to be significantly larger during task engagement when compared to non-engaged states (one-sided Wilcoxon signed rank test, *p* = 4.6×10^−4^, Figure 4C). This result was specific to the theta band, as it was not observed in either the 4-Hz (one-sided Wilcoxon signed rank test, *p* = 0.38, Figure 4D) or during gamma oscillations (one-sided Wilcoxon signed rank test, *p* = 0.53, Figure 4E). Interestingly, theta phase coherence was larger upon the application of TNP-ATP when compared to control animals (two-sided Wilcoxon signed rank test, *p* < 4.6×10^−3^, Figures 4F,G), thus suggesting that the coordination between hippocampus and prefrontal cortex was stronger upon blocking purinergic transmission. We also recorded a group of animals with electrodes located in the posterior parietal cortex, which allowed us to compute the coordination between cortical regions. Neural coupling between prefrontal and posterior parietal cortex was not affected during task execution (Figure S6), thus enhanced inter-regional coordination seemed to be specific for the hippocampus and prefrontal cortex axis.

**Figure 5:**
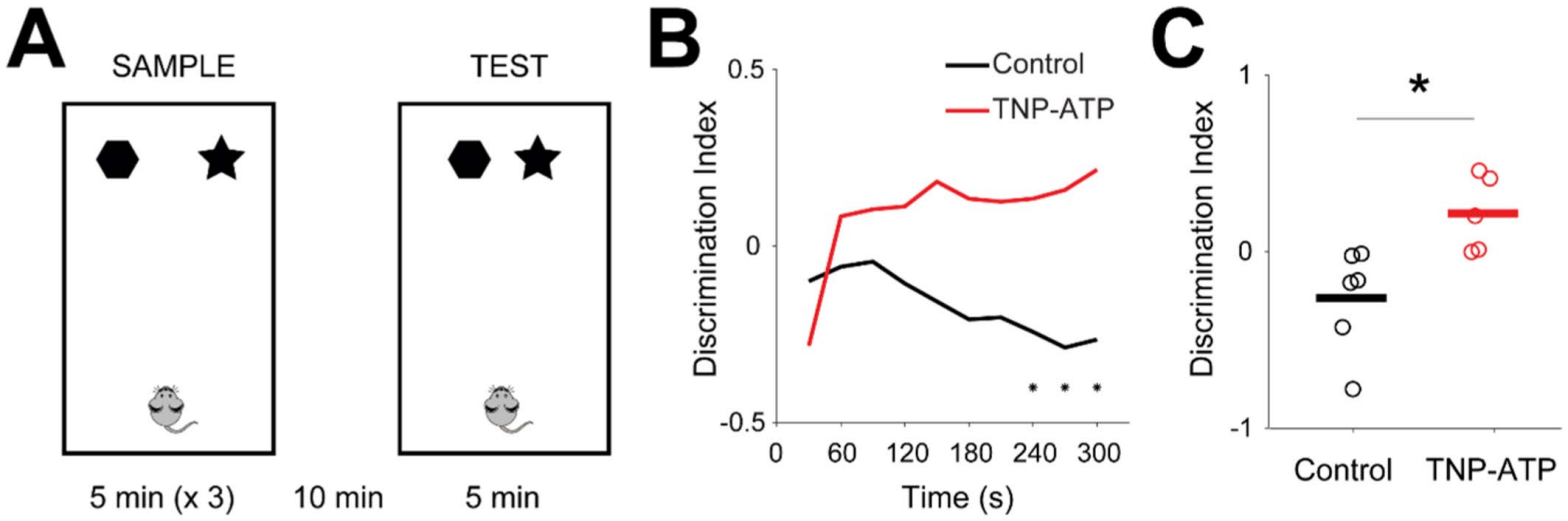
Spatial memory is rescued following blockade of purinergic receptors. **(A)** Diagram of the metric spatial change task. Mice were allowed to explore an arena containing two different objects, which were then positioned closer. **(B)** Discrimination index (ratio between the difference and the sum of exploration times of objects in the test trial and sample trial) in relation to time in the arena for control (black) and treated (red) animals (one-sided Wilcoxon signed rank test with false discovery rate correction for multiple comparisons, *p* < 0.03). **(C)** Average discrimination index for control (black) and treated (red) animals. Circles and lines represent individual mice and population averages, respectively [unpaired t-test, *t*(9) = −3.03, *p* = 0.014; −0.2 ± 0.11, control; 0.22 ± 0.09, TNP-ATP]. Asterisks point to statistically significant differences.

Finally, we further investigated behavioral performance in a spatial memory task. In this task, metric spatial change task (Kannangara et al., 2015), mice were trained to explore an arena with two objects. They then re-explored the same environment with the objects slightly displaced (Figure 5A). During the test trial, animals typically exhibit increased exploration of the objects if they detect changes in the environment, compared to the sample trial. This increase, measured by the discrimination index, serves as an indicator of hippocampus-dependent spatial memory. We observed that control animals did not recognize the displacement of objects in the test trial (Figure 5B). In contrast, treated mice showed a positive discrimination index, indicating that spatial memory was partially improved in relation to control mice (Figure 5C). These findings suggest that TNP-ATP may partially restore hippocampal function in a murine model of epilepsy, yet future studies (including control wild-type mice) are necessary to better understand the extent to which hippocampal function is normalized. At the end of experimental procedures, we exposed animals to an open field to assess their spontaneous behavior. General motor function seemed to be similar between control and treated animals, as their covered distance in the arena was not significantly different (Figure S7). Similarly, markers of anxiety, such as the time spent at the walls or number of fecal boli, were not significantly different between groups (Figure S7), thus suggesting that purinergic blockade does not affect motor function or anxiety levels in KA-injected animals.

## 4 Discussion

The pursuit of novel targetable pathological mechanisms underlying temporal lobe epilepsy is driven by the inadequacy of current antiepileptic drugs to control seizures in about one third of patients and their lack of impact on the underlying pathophysiology (Bialer and White, 2010; Pitkänen and Lukasiuk, 2011; Kobow et al., 2012). Among several proposals, altered purinergic signaling has accumulated evidence as a potential mechanism in epilepsy (Di Virgilio et al., 2023; Engel, 2023). Indeed, after injury to the central nervous system, ATP is massively released, enough as to activate the low-affinity P2XRs (Illes, 2020). The role of the P2XRs is versatile, as initially, their activation may contribute positively to neuroinflammation by triggering microglia activation and inflammasome-mediated IL-1 release, among other effects, as previously described (Roth et al., 2014; Sperlágh and Illes, 2014). However, this benefit is countered by the potential detrimental effects of aberrant P2XR expression, which can sustain neuroinflammation, induce neuronal death, and promote hyperexcitability (Murphy et al., 2012; Franceschini et al., 2015). Previous research has identified overexpression of P2X7Rs in epilepsy, but uncertainties persisted regarding the specific cell types involved and the functional role of the receptor. The potential for targeting P2XR has been explored predominantly in models of acute status epilepticus (Doná et al., 2009; Kim and Kang, 2011; Jimenez-Pacheco et al., 2013). Nevertheless, the cognitive effects of purinergic signaling in epilepsy remain little studied. This gap in knowledge underscores the need for further investigation into the role of purinergic receptors in epilepsy and their potential as a therapeutic target.

Here, we studied the influence of purinergic receptor signaling on epileptiform activity and cognitive function in a murine model of mesial temporal lobe epilepsy, prompted by KA injection in the dorsal CA1 area. We targeted P2XRs using TNP-ATP, a selective purinergic receptor antagonist, and examined behavioral performance and neural activity patterns. Our results show that TNP-ATP treatment had little effect on motor function or anxiety levels, yet it reduced both the amplitude and incidence of hippocampal epileptiform activity. Neuronal spiking associated with interictal activity was also depressed. Furthermore, hippocampus-dependent spatial memory and prefrontal cortex-dependent executive function were partially restored when behaviorally assessed. These results support the potential role of purinergic receptor antagonists in improving behavioral and cognitive performance in epilepsy, suggesting novel insight into the use of these pharmacological agents as a therapeutic approach.

While our study demonstrates promising effects of TNP-ATP on seizure reduction and cognitive function, we acknowledge the complexities associated with the timing and duration of treatment. Indeed, the ED50 of TNP-ATP varies across P2X receptor subtypes, with high potency at P2X1 receptors (ED50 around 1 nM) but lower affinity for P2X7 receptors (ED50 in the micromolar range) (Bianchi et al., 1999). Based on these pharmacodynamic properties, the plasma concentration of TNP-ATP 24 h after administration is likely below the effective threshold for receptor blockade. This raises the possibility that the observed effects reflect a combination of direct receptor blockade and indirect compensatory mechanisms triggered by prolonged receptor inhibition. These compensatory effects likely develop from the first day of treatment, as TNP-ATP was administered daily beginning 1 day before the kainic acid lesion. To address this limitation, future studies should disentangle these direct and indirect effects, to establish the potential contribution of compensatory mechanisms in shaping the observed outcomes, recognizing their potential influence on epileptiform activity, neuroinflammation, and cognitive function.

Past advancements in understanding the role of purinergic signaling, particularly the involvement of P2X7Rs, have highlighted their significance in the inflammation and progression of epilepsy (Engel et al., 2016; Fischer et al., 2016; Beamer et al., 2017). Notably, P2X7R antagonists, while showing limited efficacy in acute anticonvulsant tests and in fully kindled rats as standalone treatments, demonstrated enhanced anticonvulsant effects when used in conjunction with antiepileptic drugs (Fischer et al., 2016). Our study did not focus explicitly on quantifying anticonvulsant effects, yet we observed a marked reduction in both the amplitude and frequency of interictal discharges. This suggests a significant anticonvulsant potential for purinergic receptor antagonists even when administered independently. The discrepancies between our findings and previous studies could be attributed, at least partly, to differences in the epilepsy models used and the timing of drug administration. Indeed, our research model is induced by a single intrahippocampal injection of kainic acid (Lévesque and Avoli, 2013), which gradually evolves into an epilepsy model, and immediately initiated treatment with TNP-ATP. Contrastingly, Fischer et al. (2016) utilized a combination of pentylenetetrazol and electrical kindling to induce epilepsy and applied purinergic blockers only post-epilepsy induction (Fischer et al., 2016). Hence, in our model, the epileptiform activity had not fully developed into status epilepticus at the time of applying the purinergic receptor antagonist. Furthermore, our results align with previous findings indicating seizure reduction and neuroprotection in a murine model of epilepsy induced by intra-amygdalar kainic acid injection (Engel et al., 2012; Jimenez-Pacheco et al., 2016). This consistency underscores the potential of purinergic receptor antagonists in epilepsy treatment, warranting further investigation.

As explained, we also evaluated cognitive function. For this, we used the metric spatial change task (Kannangara et al., 2015) that assesses spatial memory. This test is particularly sensitive to spatial pattern separation, the brain’s capacity to discriminate between similar, overlapping spatial representations and store them orthogonally, which largely resides in the dentate gyrus (Yassa and Stark, 2011). Adult neurogenesis, taking place in the dentate gyrus, is remarkably important for pattern separation (Clelland et al., 2009; Sahay et al., 2011) and we have previously shown that it is severely disrupted in epilepsy (Sierra et al., 2015; Muro-García et al., 2019). Hence, it was plausible to expect detrimental effects of epilepsy in both pattern separation and spatial memory formation. Indeed, our results are consistent with recent data from pilocarpine-induced status epilepticus showing pattern separation memory impairment (Choi et al., 2024). Moreover, our findings are consistent with the efficacy of pharmacological methods using valproate or endoneuraminidase to inhibit seizure-induced neurogenesis, which not only preserves hippocampal spatial memory but also rescues features of aberrant hippocampal neurogenesis (Jessberger et al., 2007; Pekcec et al., 2008).

The impact of epilepsy on temporal lobe function, particularly in the consolidation of episodic memories mediated by the hippocampus, has been well documented (Schwarcz and Witter, 2002; Helmstaedter and Kockelmann, 2006). Similarly, the integrity of the hippocampus is crucial for spatial memory, which aligns with our results in the metric test, corroborating previous studies (O’Keefe and Nadel, 1978; Moser et al., 2017). Additionally, recent studies are increasingly linking epilepsy with aspects of executive function, notably cognitive flexibility, which depends on the activity of the medial prefrontal cortex (Hermann et al., 2002; Helmstaedter and Witt, 2017). In our study, we observed that blocking purinergic transmission in a rodent model of epilepsy affected executive function. Although the treatment did not directly enhance task efficiency, measured by the ratio of rewards collected to trial attempts, nor did it alter the rate of alternation in nose poking, it significantly improved proactive nose poking behaviors and decreased the latency to collect rewards. These findings suggest enhanced task engagement upon purinergic receptor antagonism.

Importantly, the directionality and causality within the hippocampal-prefrontal cortex axis during task performance warrant further consideration. Enhanced theta- and gamma-band coordination between these regions, as observed in kainite-injected mice, may reflect a pathological enhancement of physiological directionality of information flow during specific phases of learning and memory retrieval (Broggini et al., 2016), which is decreased by our pharmacological treatment. This aligns with previous findings, which suggest that hippocampal ventral output to the prefrontal cortex is critical for retrieving stored information (de Mooij-van Malsen et al., 2023). Such restored coordination might underlie the observed improvements in behavioral performance and cognitive function in treated animals. While our study did not explicitly separate learning and retrieval phases, and our recordings were performed in the dorsal CA1 area, the data hints at the potential for TNP-ATP to facilitate these processes, a topic for further exploration in future studies.

Cortical regions are functionally connected during multiple behavioral states. The medial prefrontal cortex expresses dominant delta oscillations which are modulated by task demands and by hippocampal inputs. Indeed, there is abundant evidence of the modulation of prefrontal gamma activity by hippocampal oscillations (Siegel et al., 2012; Roux and Uhlhaas, 2014; Backus et al., 2016). In the reverse direction, the phase of cortical delta rhythms also modulates the amplitude of hippocampal gamma oscillations. Interestingly, our results show that blocking purinergic transmission was able to restore the coupling between slow cortical rhythms and fast hippocampal oscillations. Nonetheless, we found no effect in the opposite case. Moreover, we reproduced the previously described enhanced directionality in epilepsy of the theta-range activity from the dorsal hippocampus to the medial prefrontal cortex, as computed by Granger causality (Broggini et al., 2016). In our experiments, such causality was significantly decreased by purinergic receptor antagonism; yet, the opposite directionality, from the medial prefrontal cortex to the hippocampus was not modified by treatment. Importantly, our pharmacological treatment was able to restore the functional hippocampo-cortical connectivity selectively in the theta band during task execution, which has been previously demonstrated to be relevant for efficient task performance (Roux and Uhlhaas, 2014; Backus et al., 2016). These results suggest that output modulatory projections from the hippocampus are disrupted in the epilepsy model, but not input modulatory afferences, such as those arising from the medial prefrontal cortex. Additionally, blocking purinergic receptors seems to partially restore the functional connectivity in the hippocampo-cortical axis. Overall, our results monitoring both the behavioral and functional connectivity impacts contributes to a substantiate the use of purinergic antagonists in epilepsy’s pathophysiology and potential new treatment avenues.

Despite demonstrating that blocking purinergic signaling with TNP-ATP reduces seizure severity and improves behavioral outcomes, our findings must be interpreted in the context of several limitations. The overarching descriptor of purinergic receptor antagonism oversimplifies the complexity of purinergic signaling. Indeed, while we note that TNP-ATP inhibits P2X receptors, it is important to identify which of the seven P2X subtypes and potentially other purinergic receptors are involved. The purinergic system encompasses P2X, P2Y, and P1 receptors, each with distinct roles in neurotransmission and pathology; therefore, future studies will have to address a more precise identification of the receptor subtypes blocked for understanding the mechanisms behind our results.

Moreover, our study examined the acute effects of kainate, which induces seizures rather than chronic epilepsy, where spontaneous recurrent seizures are observed. Although we refer to epilepsy in our study, the present experiments primarily capture the acute seizure phase. In subsequent studies, it will be important to determine whether mice actually transition to a state of chronic epilepsy and to clarify when TNP-ATP is administered relative to both acute and potential chronic seizure episodes. Similarly, the extent to which TNP-ATP’s effects on behavior are directly linked to its seizure-suppressing properties remains unresolved. While our data indicate that mice treated with TNP-ATP perform better in behavioral tests, correlating the degree of seizure reduction with behavioral outcomes would more definitively establish whether the improved behavior results primarily from decreased seizure severity or from additional neuroprotective or anti-inflammatory actions of TNP-ATP.

Finally, the pharmacokinetics of TNP-ATP in the brain, including its half-life and clearance, remains insufficiently characterized. Without confirming that TNP-ATP is still active at the time of kainate-induced seizures and behavioral testing, it is difficult to assert that all observed effects are indeed attributable to purinergic receptor blockade. Future studies employing more precise pharmacokinetic measurements, and possibly alternative, receptor-specific ligands, would clarify the duration and specificity of the action of TNP-ATP, thus strengthening our conclusions. By addressing these limitations, it will be possible to build a future stronger foundation for the therapeutic potential of purinergic antagonists in seizure-related disorders.

## Data Availability

The raw data supporting the conclusions of this article will be made available by the authors, without undue reservation upon reasonable request.

## Footnotes

^1^ https://users.sussex.ac.uk/~lionelb/MVGC/html/mvgchelp.html#1

^2^ http://fieldtrip.fcdonders.nl/

^3^ http://www.chronux.org
