# Supplementary material for: Purinergic receptor antagonism reduces interictal discharges and rescues cognitive function in a mouse model of temporal lobe epilepsy
Source: Front Neurosci. 2025 Apr 4;19:1513135. doi: 10.3389/fnins.2025.1513135 (PMC12007451; doi:10.3389/fnins.2025.1513135)
Supplement: Supplementary file 1 [file Data_Sheet_1.docx]

**Supplementary Figures**


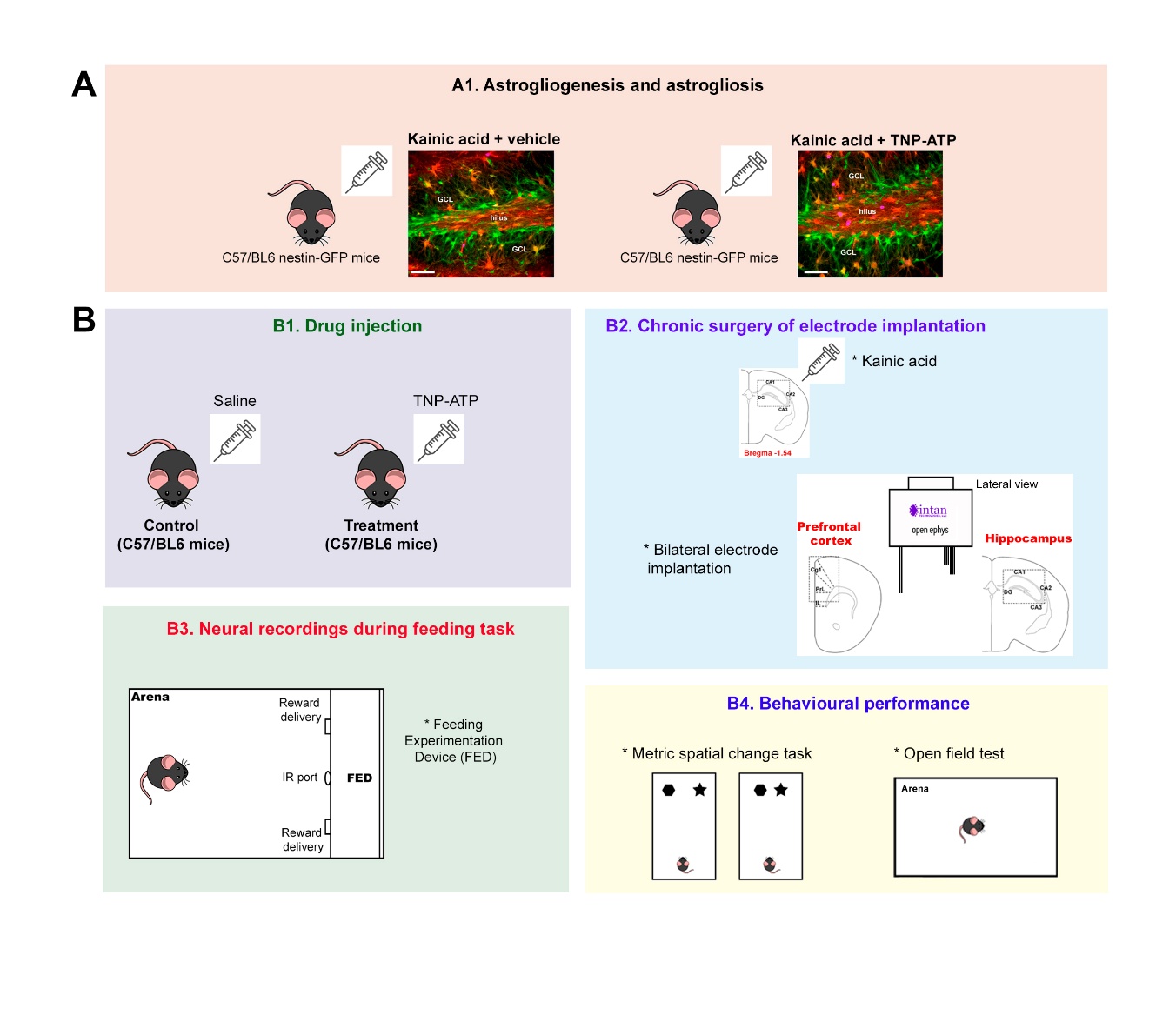


**Figure S1.** Experimental design. A, scheme depicting the effects of TNP-ATP on astrogliogenesis and astrogliosis in the dentate gyrus. A1, quantitative analysis shows that treated mice exhibited a reduced number of both astrocytes and reactive astrocytes compared to controls. B, chronic recordings and behavioral experiments. B1, one day after surgery, C57/BL6 mice were randomized and injected with either saline or TNP-ATP. B2, surgical procedures: animals in both the treatment and control groups were first injected with kainic acid in the hippocampus, followed by the bilateral implantation of electrode arrays in the prefrontal cortex (PFC) and hippocampus, and then a recovery period of 7 days. B3, cognitive function assessment in the epilepsy model, where neural activity in the PFC-hippocampus circuit was recorded during the feeding task using the FED for behavioral analysis, spectral analysis, synchrony analysis (cross-frequency modulation), and Granger causality analysis. B4, assessing performance in behavioral tests, which included a metric test to evaluate spatial memory tasks and an open field test to assess spontaneous behavior and anxiety levels in both the treatment and control groups.


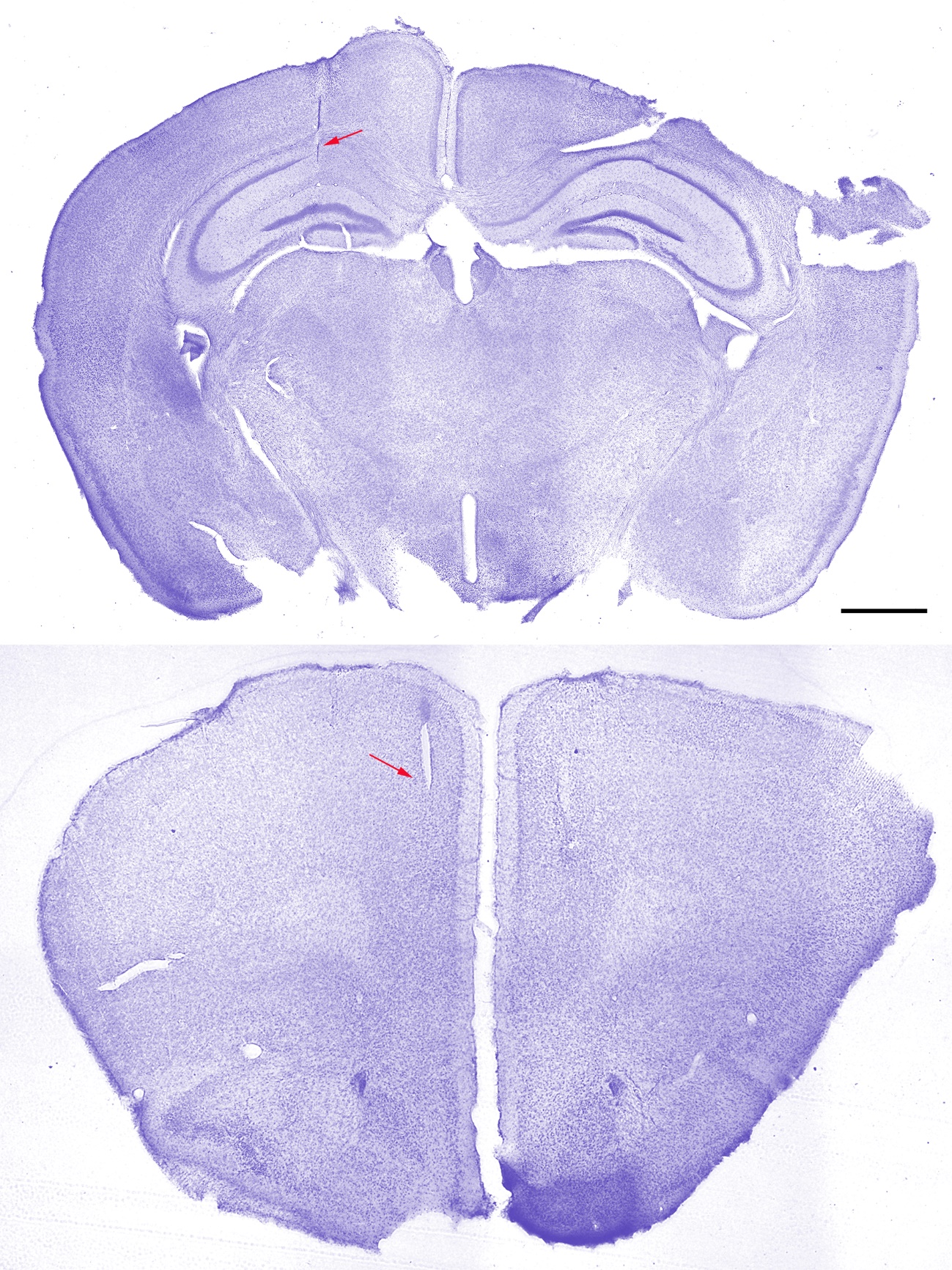


**Figure S2.** Example recording sites. Nissl-stained coronal sections depict brain lesions (red arrows) showing the location of the implanted recording electrodes in the hippocampus (top, dorsal CA1 area) and prefrontal cortex (bottom, prelimbic area). Scalebar, 1 mm.


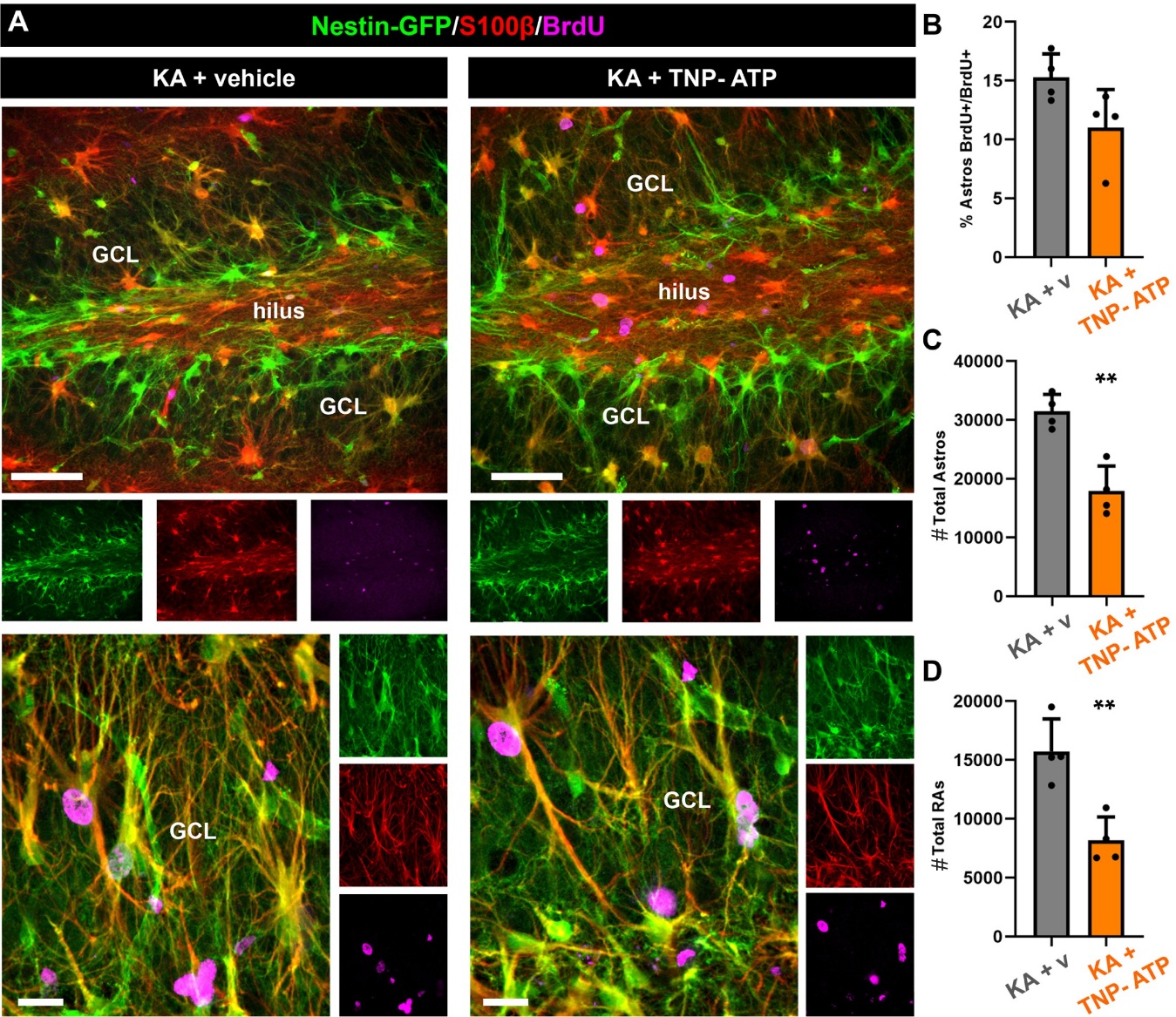


**Figure S3.** A, representative confocal images of brain slices form KA-injected Nestin-GFP mice, treated with either vehicle or TNP-ATP after immunostaining for GFP, S100β and BrdU, at lower (upper panels, scale bar 50 µm) and higher (lower panels, scale bar 10 µm) magnification. B, quantification of the proportion of dividing astrocytes, including reactive astrocytes, immunostained for BrdU in the granule cell layer (GCL) and hilus. C, number of astrocytes (immunonegative for Nestin-GFP but immunopositive for S100β in the GCL and hilus. D, Number of reactive astrocytes (immunopositive for Nestin-GFP and for S100β) in the GCL and hilus. n = 4 mice in each group. **P < 0.005 after Student´s t test. Astrocytes were differentiated from NSCs based on the expression of the specific biomarker S100β, which is only expressed by astrocytes and not NSCs. Reactive astrocytes and NSCs express nestin (and Nestin-GFP) as well as GFAP. However, NSCs transform into reactive astrocytes in the experimental model of MTLE being phenotypically indistinguishable (by morphology and expression of biomarkers).


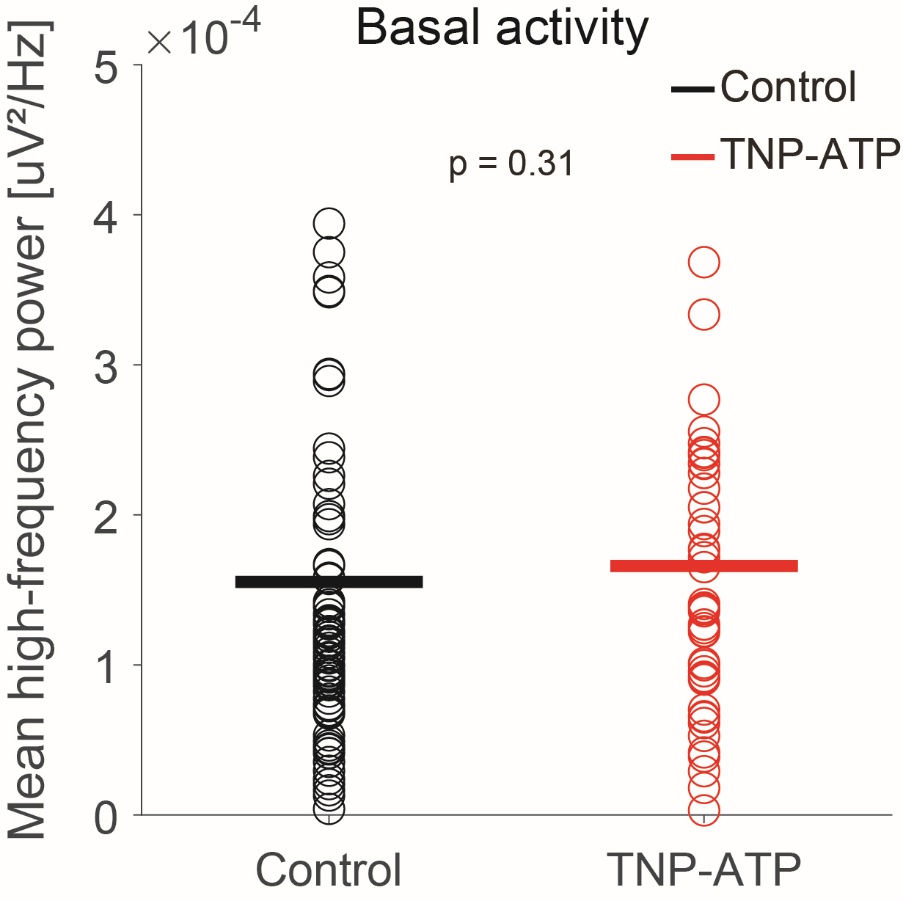


**Figure S4.** Power of baseline high-frequency activity in the hippocampus. Left, all trials (one-sided Wilcoxon signed rank test, z = 18.3, P = 5.18x10^-75^). Right, randomly selected within 2 z-score of the FED mean speed, (one-sided Wilcoxon signed rank test, n = 552, z = 0.05, P = 0.96).

**
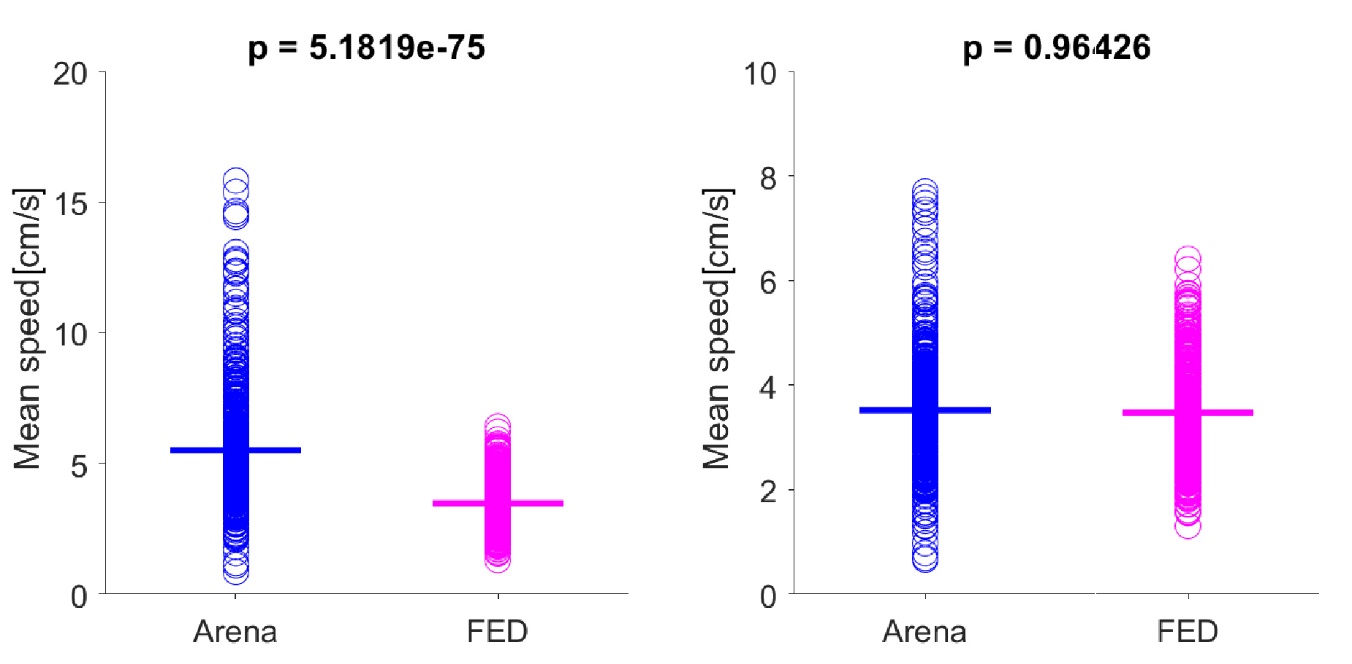
Figure S5.** Locomotor speed in the FED task. Left, all trials (one-sided Wilcoxon signed rank test, z = 18.3, P = 5.18x10^-75^). Right, randomly selected within 2 z-score of the FED mean speed, (one-sided Wilcoxon signed rank test, n = 552, z = 0.05, P = 0.96).

**
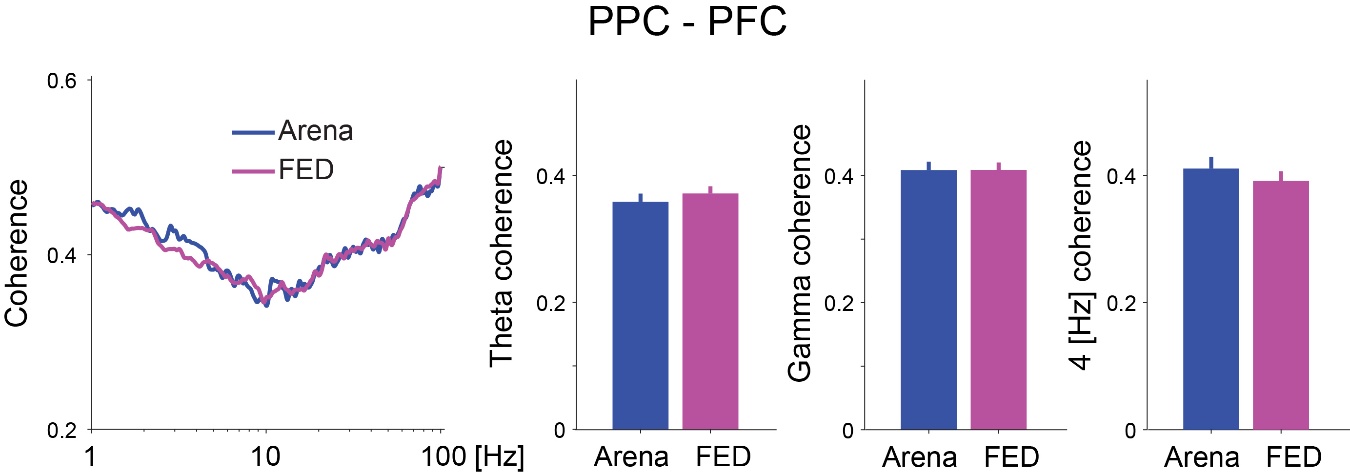
**

**Figure S6.** Phase coherence between hippocampus and posterior parietal cortex during FED performance (n = 4 mice). One-sided Wilcoxon signed rank test; theta coherence, z = -1.13, P = 0.26; gamma coherence, z = -1.03, P = 0.30; 4-Hz coherence, z = 1.3, P = 0.19.


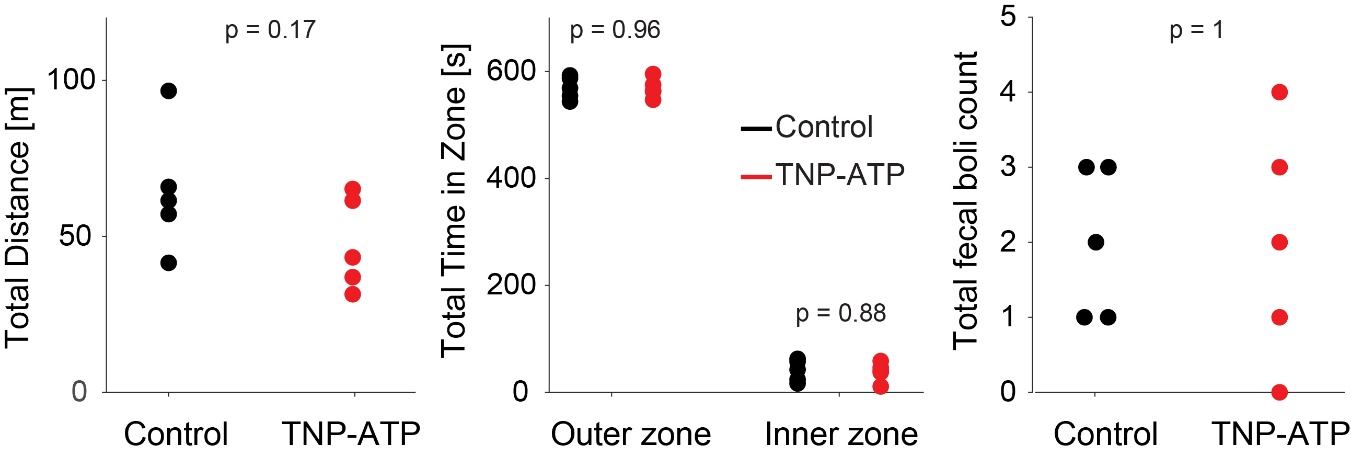


**Figure S7.** Locomotor activity and anxiety behaviour. Total distance: unpaired t-test, P = 0.17, t(8) = 1.5; Total time in outer zone: unpaired t-test, P = 0.96, t(8) = 0.05; Total time in inner zone: unpaired t-test, P = 0.88, t(8) = 0.16; Total fecal boli count, P = 1, t(8) = 0.

**Supplementary table 1**

| **Statistical analysis** | **Variable** | **Comparison group** | **Figure** | **Standardized test statistic value** | **Degrees of freedom** | **p-value** | **p-value for normality test (*)** | **Conclusion** |
| --- | --- | --- | --- | --- | --- | --- | --- | --- |
| Wilcoxon rank-sum test | PSD of interictal discharges | Control vs. TNP-ATP | 1C | z = 2.4 | N/A | 0.015 | p1=1.2x10-17 p2=7.2x10-11 | Significant difference |
| Unpaired t-test | Interictal activity propagation | Control vs. TNP-ATP | 1D | t = 2.2 | df = 47 | 0.031 | p1=2.1x10-31 p2=1.3x10-14 | Significant difference |
| Unpaired t-test | Interictal discharge amplitude | Control vs. TNP-ATP | 1E | t = 2.5 | df = 47 | 0.012 | p1=1.9x10-9 p2=1.3x10-4 | Significant difference |
| Unpaired t-test | Interictal activity density | Control vs. TNP-ATP | 1F | t = 2.3 | df = 158 | 0.019 | p1=1.8x10-20 p2=4.4x10-16 | Significant difference |
| Unpaired t-test | Interictal discharge duration | Control vs. TNP-ATP | 1G | t = 0.46 | df = 120 | 0.64 | p1=4.4x10-21 p2=5.3x10-13 | Non-significant difference |
| Unpaired t-test | Firing rate | Control vs. TNP-ATP | 1H | t = 0.39 | df = 40 | 0.69 | p1=1.6x10-17 p2=5.0x10-14 | Non-significant difference |
| Unpaired t-test | Coupling of neuronal spiking before interictal discharges | Control vs. TNP-ATP | 1I | t = -2.6 | df = 81 | 0.012 | p1=1.0x10-28 p2=1.0x10-14 | Significant difference |
| Unpaired t-test | Coupling of neuronal spiking after interictal discharges | Control vs. TNP-ATP | 1I | t = 2.6 | df = 81 | 0.012 | p1=2.9x10-31 p2=2.9x10-21 | Significant difference |
| One-way ANOVA | Locomotion speed changes | Control vs. TNP-ATP vs. pre- vs. post- Ictal discharge | 1K | F = 13.36 | df(groups) = 3 df(error) = 122 | 1.34x10-7 | p1=3.7x10-15 p2=2.2x10-8 | Significant difference |
| Pearson correlation | Number of nose-pokes (Control) | # nose pokes - day | 2B | r = 0.95 | N/A | 3.5x10-4 | N/A | Significant correlation |
| Pearson correlation | Number of nose-pokes (TNP-ATP) | # nose pokes - day | 2B | r = 0.93 | N/A | 7.5x10-4 | N/A | Significant correlation |
| Bootstrap test | Nose pokes increase rate | Control vs. TNP-ATP | 2B | N/A | N/A | 5x10-4 | N/A | Significant difference |
| Pearson correlation | Collected reward number (Control) | Collected pellet - day | 2C | r = 0.98 | N/A | 4.9x10-5 | N/A | Significant correlation |
| Pearson correlation | Collected reward number (TNP-ATP) | Collected pellet - day | 2C | r = 0.92 | N/A | 1.3x10-3 | N/A | Significant correlation |
| Bootstrap test | Collected reward number increase rate | Control vs. TNP-ATP | 2C | N/A | N/A | 0.02 | N/A | Significant difference |
| Pearson correlation | Latency for pellet collection (Control) | Latency - day (TNP-ATP) | 2D | r = -0.88 | N/A | 3.1x10-3 | N/A | Significant correlation |
| Pearson correlation | Latency for pellet collection (TNP-ATP) | Latency - day (TNP-ATP) | 2D | r = -0.8 | N/A | 0.013 | N/A | Significant correlation |
| Bootstrap test | Latency for pellet collection rate | Control vs. TNP-ATP | 2D | N/A | N/A | 10-4 | N/A | Significant difference |
| Pearson correlation | Task efficiency (Control) | Efficiency - day | 2E | r = 0.35 | N/A | 0.35 | N/A | Non-significant correlation |
| Pearson correlation | Task efficiency (TNP-ATP) | Efficiency - day | 2E | r = 0.61 | N/A | 0.08 | N/A | Non-significant correlation |
| Pearson correlation | Alternance proportion (Control) | Alternance % - day | 2F | r = 0.43 | N/A | 0.24 | N/A | Non-significant correlation |
| Pearson correlation | Alternance proportion (TNP-ATP) | Alternance % - day | 2F | r = -0.6 | N/A | 0.09 | N/A | Non-significant correlation |
| Wilcoxon rank-sum test | PFC power spectral density (4 Hz) | Control vs. TNP-ATP | 3C | z = 3.5 | N/A | 4.0x10-4 | p1=5.0x10-15 p2=4.1x10-8 | Significant difference |
| Wilcoxon rank-sum test | PFC crossfrequency modulation | Control vs. TNP-ATP | 3E | z = -2.57 | N/A | 0.01 | p1=2.0x10-15 p2=3.8x10-5 | Significant difference |
| Wilcoxon rank-sum test | Ascending Granger causality (CA1 -> PFC) | Control vs. TNP-ATP | 3F | N/A | N/A | < 0.001 | p1 < 0.05 p2< 0.05 | Significant difference |
| False discovery rate for Wilcoxon signed rank | Ascending Granger causality (CA1 -> PFC) | Control vs. TNP-ATP | 3F | N/A | N/A | < 0.001 | N/A | Significant difference |
| Wilcoxon signed-rank test | Granger causality magnitude (theta) | Control vs. TNP-ATP | 3G | z = 3.5 | N/A | 3.3x10⁻⁵ | p1=3.5x10-100 p2=2.3x10-47 | Significant difference |
| Wilcoxon rank-sum test | Granger causality magnitude (gamma) | Control vs. TNP-ATP | 3H | z = 3.3 | N/A | 8.2x10-4 | p1=3.4x10-100 p2=2.3x10-47 | Significant difference |
| False discovery rate for Wilcoxon signed rank | Phase coherence (CA1 - PFC ) | Arena vs. FED | 4B | N/A | N/A | < 0.004 | N/A | Significant difference |
| Wilcoxon signed-rank test | Phase coherence (theta, CA1-PFC) | Arena vs. FED | 4C | z = -3.5 | N/A | 4.6x10-4 | p1=5.5x10-21 p2=3.1x10-27 | Significant difference |
| Wilcoxon signed-rank test | Phase coherence (4Hz, CA1-PFC) | Arena vs. FED | 4D | z = -0.87 | N/A | 0.38 | p1=9.2x10-22 p2=2.8x10-27 | Non-significant difference |
| Wilcoxon signed-rank test | Phase coherence (gamma, CA1-PFC) | Arena vs. FED | 4E | z = -0.63 | N/A | 0.53 | p1=2.6x10-23 p2=1.8x10-27 | Non-significant difference |
| False discovery rate for Wilcoxon signed rank | Phase coherence (CA1 - PFC ) | Control vs. TNP-ATP | 4F | N/A | N/A | <4.6x10-3 | N/A | Significant difference |
| Two-way ANOVA | Phase coherence (theta,CA1 - PFC ) | Control vs. TNP-ATP vs. Arena vs. FED | 4G | F(Arena-FED)= 5.5 F(Control-TNP-ATP) = 4.7 F(interaction): 0.66 | df(total) = 155 | 0.02 | p(resid.) = 3.1x10-26 | Significant difference |
| Wilcoxon rank-sum test | High frequency basaline activity | Control vs. TNP-ATP | S4 | z = -1.02 | N/A | 0.31 | p1=2.1x10-17 p2=8.2x10-11 | Non-significant difference |
| Wilcoxon signed-rank test | Speed | Arena vs. FED | S5 | z = 18.3 | N/A | 5.18x10-75 | p1≈0 p2≈0 | Significant difference |
| Wilcoxon signed-rank test | Speed restricted to 2 z-score | Arena vs. FED | S5 | z = 0.05 | N/A | 0.96 | p1≈0 p2≈0 | Non-significant difference |
| Wilcoxon signed-rank test | Phase coherence (theta, PPC-PFC) | Arena vs. FED | S6 | z = -1.13 | N/A | 0.26 | p1=6.2x10-17 p2=1.8x10-20 | Non-significant difference |
| Wilcoxon signed-rank test | Phase coherence (gamma, PPC-PFC) | Arena vs. FED | S6 | z = -1.03 | N/A | 0.3 | p1=1.9x10-18 p2=1.9x10-21 | Non-significant difference |
| Wilcoxon signed-rank test | Phase coherence (4Hz, PPC-PFC) | Arena vs. FED | S6 | z = 1.3 | N/A | 0.19 | p1=1.8x10-17 p2=3.2x10-20 | Non-significant difference |
| Unpaired t-test | Total distance | Control vs. TNP-ATP | S7 | t = 1.5 | df = 8 | 0.17 | p1= 0.69 p2=0.49 | Non-significant difference |
| Unpaired t-test | Outer zone time | Control vs. TNP-ATP | S7 | t = 0.05 | df = 8 | 0.96 | p1= 0.82 p2=0.97 | Non-significant difference |
| Unpaired t-test | Inner zone time | Control vs. TNP-ATP | S7 | t = 0.16 | df = 8 | 0.88 | p1= 0.66 p2=0.76 | Non-significant difference |
| Unpaired t-test | Total fecal boli count | Control vs. TNP-ATP | S7 | t = 0 | df = 8 | 1 | p1= 0.15 p2=1 | Non-significant difference |

(*) n > 10: Kolmogórov-Smirnov test; n < 10: Shapiro-Wilk
